# COVID-19-related stress, exercise, and oral health-related quality of life among community-dwelling older adults who participated in the CHEER Iwamizawa project, Japan

**DOI:** 10.1038/s41598-022-24806-1

**Published:** 2022-11-27

**Authors:** Kazuhito Miura, Yutaka Watanabe, Haruhisa Baba, Kimiya Ozaki, Takae Matsushita, Miyako Kondoh, Kazutaka Okada, Shinji Nakaoka, Katsuhiko Ogasawara, Teppei Suzuki, Hiroshi Saito, Takashi Kimura, Akiko Tamakoshi, Yutaka Yamazaki

**Affiliations:** 1 Gerodontology, Department of Oral Health Science, Faculty of Dental Medicine, Hokkaido University, Kita 13 Nishi 7, Kita-Ku, Sapporo City, Hokkaido, 060-8586 Japan; 2 Laboratory of Mathematical Biology, Department of Advanced Transdisciplinary Sciences, Faculty of Advanced Life Science, Hokkaido University, Kita 10 Nishi 8, Kita-Ku, Sapporo City, Hokkaido, 060-0810 Japan; 3 Health Innovation and Technology Center, Faculty of Health Sciences, Hokkaido University, Kita 12 Nishi 5, Kita-Ku, Sapporo City, Hokkaido, 060-0812 Japan; 4 Hokkaido University of Education Iwamizawa Campus, 2-34 Midorigaoka, Iwamizawa City, Hokkaido, 068-8642 Japan; 5 Department of Public Health, Faculty of Medicine, Hokkaido University, Kita 15 Nishi 7, Kita-Ku, Sapporo City, Hokkaido, 060-8638 Japan

**Keywords:** Public health, Dentistry, Risk factors, Epidemiology, Lifestyle modification

## Abstract

This study examined the association between coronavirus disease 2019 (COVID-19)-related stress, exercise habits, and oral health-related quality of life (OHRQoL) in a sample of 215 community-dwelling older adults in Japan (57 men, 158 women; *M*_age_ = 74.2 years, *SD* = 6.0). Data were collected during wellness checkups in October 2020 and included participants’ demographic characteristics, measures of instrumental activities of daily living and depressive tendencies, number of teeth, oral hypofunction, OHRQoL, COVID-19-related stress, and exercise habits. Four mutually exclusive groups were created, using the presence or absence of COVID-19-related stress and lack of exercise habits as risk factors for poor OHRQoL (no COVID-19-related stress and no lack of exercise, COVID-19-related stress only, lack of exercise habits only, and both COVID-19-related stress and lack of exercise habits). Poisson regression with robust standard errors provided the prevalence ratio for poor OHRQoL. The presence of both COVID-19-related stress and lack of exercise habits (adjusted prevalence ratio: 2.20, 95% CI: 1.31– 3.69) was associated with poor OHRQoL. The results indicate that COVID-19-related stress and exercise habits should be considered when designing oral health and public health initiatives.

## Introduction

In recent times, the prevalence of mental health problems has increased, owing to the coronavirus disease 2019 (COVID-19) pandemic^1–3^. Mobility restrictions and lifestyle changes due to the pandemic have been found to cause stress^4,5^. In addition, older adults have been reported to have a higher risk of death and exacerbation of COVID-19, making them more anxious and fearful about contracting COVID-19 than younger people^6,7^.

OHRQoL is a multidimensional construct that includes a subjective evaluation of the individual’s oral health, functional well-being, emotional well-being, expectations and satisfaction with care, and sense of self, which is an integral part of general health and well-being^8^. In the aftermath of the Great East Japan Earthquake, an increase in subjective toothache^9^ and a decrease in OHRQoL were reported by disaster victims^10^, suggesting that life-threatening crises can have a significant negative impact on oral and mental health. The COVID-19 pandemic has been predicted to have a similar impact on OHRQoL. Thus, anxiety about COVID-19 has been associated with worsening daytime teeth clenching^11^, periodontal disease^12^, and poor OHRQoL^13^.

In contrast, exercise has been found to enhance the quality of life (QoL) by reducing psychological distress^14^ and eliciting favorable effects on mental health and chronic pain control^15,16^. Although no study has examined the relationship between exercise and OHRQoL thus far, exercise may affect OHRQoL by alleviating psychological distress. The COVID-19 pandemic has led to a decrease in physical activity among community-dwelling older adults^17^. Previous research has shown that a reduction in mobility is a risk factor for increased functional disability in the future^18^.

Furthermore, poor OHRQoL has been reportedly related to general frailty and lower QoL^19–22^, thus indicating the need to maintain good subjective oral health. Therefore, the factors associated with OHRQoL should be considered when designing oral healthcare and public health programs.

We hypothesized that the stress caused by mobility restrictions and lifestyle changes during the COVID-19 pandemic (COVID-19-related stress; CS) and lack of exercise habits (LEH) would be risk factors for poor OHRQoL and that the presence of both would be associated with poor OHRQoL. Therefore, this cross-sectional study aimed to determine the association between CS, LEH, and OHRQoL in community-dwelling older adults.

## Results

### Participant characteristics

A total of 232 participants were included in the study, representing 0.7% of the citizens aged over 60 years in Iwamizawa City (see Supplemental Fig. S1). Of these, ten participants who gave incomplete answers to the questionnaire and seven who did not wish to undergo dental examinations and oral function tests were excluded. Thus, the total number of participants eligible for analysis was 215 (57 men and 158 women; *M*_age_ = 74.2 years, *SD* = 6.0).

### COVID-19-related stress

Ninety-seven participants (45.1%) were classified as having CS. No significant differences were found in their demographic characteristics and other variables according to the presence or absence of CS (Table 1).

**Table 1:** Comparisons between the two groups with and without COVID-19-related stress.

Variable	Overall (N = 215)		CS(−) (n = 118)		CS( +) (n = 97)		*P*-value
	Median[Q1,Q3] n (%)		Median[Q1,Q3] n (%)		Median[Q1,Q3] n (%)		
Age, years	74.0 [70.0, 79.0]		75.0[71.0, 78.0]		74.0[69.0, 79.0]		0.262
Women, n (%)	158	(69.8)	82	(69.5)	76	(78.4)	0.164
Current smokers, n (%)	10	(4.7)	7	(5.9)	3	(3.1)	0.517
BMI	22.7 [20.6, 25.2]		22.6[20.9, 25.3]		22.7[20.5, 24.5]		0.672
JST-IC score	13.0 [11.0, 15.0]		13.0[11.0, 15.0]		13.0[11.0, 15.0]		0.435
Medical history, n (%)							
Malignant neoplasm	32	(14.9)	17	(14.4)	15	(15.5)	0.849
Stroke	6	(2.8)	2	(1.7)	4	(4.1)	0.413
Myocardial infarction	3	(1.4)	3	(2.5)	0	(0.0)	0.254
Depression	4	(1.9)	2	(1.7)	2	(2.1)	1.000
Osteoarthritis	38	(17.7)	20	(16.9)	18	(18.6)	0.858
Depressive tendencies	23	(10.7)	10	(8.5)	13	(13.4)	0.273
Oral function							
Number of teeth	22.0 [15.0, 26.0]		22.0[15.0, 25.0]		23.0[17.0, 26.0]		0.086
TCI (%)	27.8 [11.1, 50.0]		25.0[11.1, 50.0]		33.3[11.1, 50.0]		0.832
Oral moisture	28.8 [27.5, 29.9]		28.8[27.5, 30.0]		28.9[27.5, 29.9]		0.955
Occlusal force (N)	550.3 [365.6, 825.0]		577.1[366.3, 825.0]		528.7[365.6, 794.0]		0.590
ODK (times/s)	5.8 [5.6, 6.4]		5.8[5.4, 6.2]		6.0[5.6, 6.4]		0.051
Tongue pressure (kPa)	34.9 [30.1, 38.8]		34.8[30.0, 38.8]		35.2[30.1, 39.0]		0.982
Masticatory function (mg/dL)	211.0 [172.0, 242.0]		202.0[169.5, 236.0]		214.0[177.0, 236.0]		0.766
Oral hypofunction	57	(26.5)	32	(27.1)	25	(25.8)	0.877

Categorical variables are shown as numbers (percentages) and were analyzed by performing either the chi-square test or Fisher’s exact test. Continuous variables are expressed as median [Q1, Q3], and were analyzed by performing the Mann–Whitney U test. *P*-values < 0.05 were considered statistically significant.

Abbreviations: *BMI* body mass index; *CS* COVID-19-related stress; *JST-IC* Japan Science and Technology Agency Index of Competence; *ODK* oral diadochokinesis; *Q1*, first quartile; *Q3* third quartile*; TCI* tongue coating index.

### Lack of exercise habits

Fifty-nine participants (27.4%) were classified as having LEH. The score on the Japan Science and Technology Agency Index of Competence (JST-IC) was significantly lower in the group with LEH. No significant differences were found in age, medical history, oral function, or rates of patients with oral hypofunction(Table 2).

**Table 2:** Comparison between the two groups with and without lack of exercise habits.

Variable	LEH(−) (n = 156)		LEH( +) (n = 59)		*P*-value
	Median[Q1,Q3] n (%)		Median[Q1,Q3] n (%)		
Age, years	74.0[70.0, 78.5]		74.0[70.0, 78.5]		0.956
Women, n(%)	117	(75.0)	41	(69.5)	0.489
Current smokers, n (%)	5	(3.2)	5	(8.5)	0.142
BMI	22.3[20.4, 24.2]		23.9[20.8, 26.1]		0.051
JST-IC score	13.0[11.5, 15.0]		12.0[9.5, 15.0]		0.011
Medical history, n (%)					
Malignant neoplasm	21	(13.5)	11	(18.6)	0.391
Stroke	5	(3.2)	1	(1.7)	1.000
Myocardial infarction	2	(1.3)	1	(1.7)	1.000
Depression	4	(2.6)	0	(0.0)	0.577
Osteoarthritis	27	(17.3)	11	(18.6)	0.842
Depressive tendencies	14	(9.0)	9	(15.3)	0.217
Oral function					
Number of teeth	22.0[16.0, 26.0]		22.0[12.5, 27.0]		0.752
TCI (%)	22.2[11.1, 47.2]		33.3[11.1, 61.1]		0.076
Oral moisture	28.8[27.4, 29.9]		29.0[27.9, 30.3]		0.458
Occlusal force (N)	573.5[370.9, 832.4]		458.9[283.3, 757.5]		0.312
ODK (times/s)	6.0[5.6, 6.4]		5.8[5.4, 6.2]		0.139
Tongue pressure (kPa)	34.8[29.3, 38.8]		36.1[32.4, 40.1]		0.068
Masticatory function (mg/dL)	213.0[175.0, 253.0]		205.0[169.5, 232.5]		0.149
Oral hypofunction	42	(26.9)	15	(25.4)	0.864

Categorical variables are shown as numbers (percentages) and were analyzed by performing either the chi-square test or Fisher’s exact test. Continuous variables are expressed as median [Q1, Q3], and were analyzed by performing the Mann–Whitney U test. *P*-values < 0.05 were considered statistically significant.

Abbreviations: BMI, body mass index; JST-IC, Japan Science and Technology Agency Index of Competence; LEH, Lack of exercise habits; ODK, oral diadochokinesis; Q1, first quartile; Q3, third quartile; TCI, tongue coating index.

### Risk of poor OHRQoL

**Figure 1:**
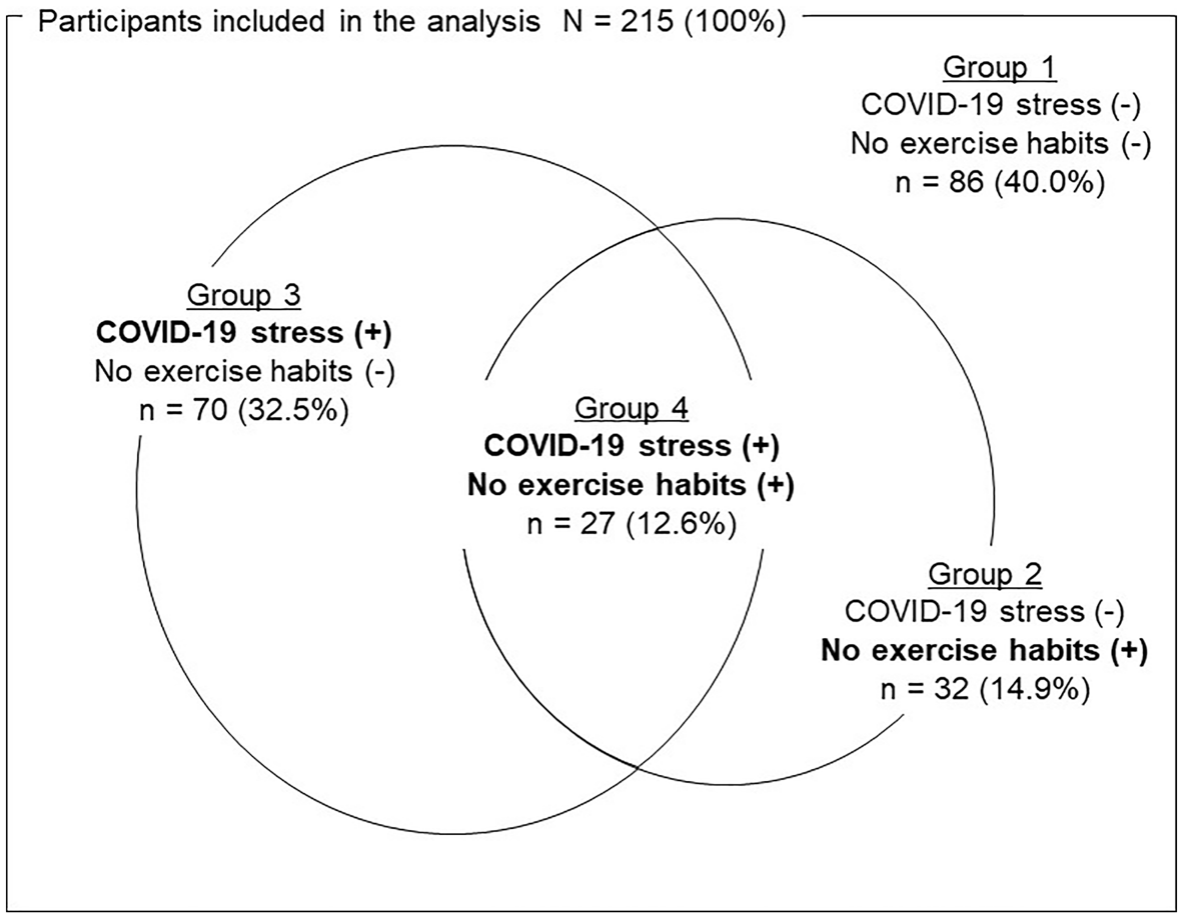
Classification of the four groups of risk of poor OHRQoL. The percentages of participants across the four groups according to the presence or absence of risk factors for poor OHRQoL are shown.

Figure 1 shows the percentages of participants across the four groups according to the presence or absence of risk factors for poor OHRQoL. Group 1 had 86 participants (40.0%), Group 2 had 32 (14.9%), Group 3 had 70 (32.5%), and Group 4 had 27 (12.6%). No significant differences were found in the rate of oral hypofunction and other variables in each group (Table 3).

**Table 3:** Comparisons among the four groups for the risk of poor OHRQoL.

Variable	Group 1: CS(-), LEH(−) (n = 86)		Group 2: CS(−), LEH( +) (n = 32)		Group 3: CS( +), LEH(−) (n = 70)		Group 4: CS( +), LEH( +) (n = 27)		*P*-value
	Median[Q1,Q3] n (%)		Median[Q1,Q3] n (%)		Median[Q1,Q3] n (%)		Median[Q1,Q3] n (%)		
Age, years	74.5[71.0, 78.0]		75.0[70.0, 78.0]		73.0[69.0, 79.0]		74.0[69.0, 81.0]		0.655
Women, n(%)	60	(69.8)	22	(68.8)	57	(81.4)	19	(70.4)	0.347
Current smokers, n (%)	4	(4.7)	3	(9.4)	1	(1.4)	2	(7.4)	0.185
BMI	22.3[20.4, 24.1]		25.3[21.2, 26.5]		22.7[20.5, 24.4]		22.7[20.6, 25.2]		0.112
JST-IC score	14.0[11.0, 15.0]		13.0[9.0, 15.0]		12.0[10.0, 14.5]		13.0[12.0, 15.0]		0.072
Medical history, n (%)									
Malignant neoplasm	12	(14.0)	5	(15.6)	9	(12.9)	6	(22.2)	0.713
Stroke	1	(1.2)	1	(3.1)	4	(5.7)	0	(0.0)	0.318
Myocardial infarction	2	(2.3)	1	(3.1)	0	(0.0)	0	(0.0)	0.499
Depression	2	(2.3)	0	(0.0)	2	(2.9)	0	(0.0)	1.000
Osteoarthritis	14	(16.3)	6	(18.8)	13	(18.6)	5	(18.5)	0.961
Depressive tendencies	4	(4.7)	6	(18.8)	10	(14.3)	3	(11.1)	0.066
Oral function									
Number of teeth	22.0[15.0, 25.0]		22.0[12.5, 25.5]		23.0[18.0, 26.0		23.0[13.5, 27.0]		0.380
TCI(%)	16.7[11.1, 50.0]		33.3[13.9, 61.1]		25.0[11.1, 44.4]		33.3[8.3, 55.6]		0.346
Oral moisture	28.8[27.4, 29.9]		28.9[27.7, 30.4]		28.9[27.4, 29.9]		29.1[28.1, 30.0]		0.906
Occlusal force (N)	603.2[374.3, 825.0]		496.5[298.2, 825.4]		531.8[367.0, 839.8]		454.5[267.5, 647.5]		0.722
ODK (times/s)	5.8[5.5, 6.2]		5.8[5.2, 6.0]		6.0[5.6, 6.4]		5.8[5.4, 6.3]		0.109
Tongue pressure (kPa)	34.2[29.3, 38.7]		36.8[33.0, 40.3]		35.3[28.9, 39.0]		35.2[31.5, 39.4]		0.286
Masticatory function (mg/dL)	207.0[165.0, 257.0]		202.0[169.5, 236.0]		215.0[178.0, 249.0]		211.0[170.5, 231.5]		0.475
Oral hypofunction	24	(27.9)	8	(25.0)	18	(25.7)	7	(25.9)	0.987

Categorical variables are shown as numbers (percentages) and were analyzed by performing either the chi-square test or Fisher’s exact test. Continuous variables are expressed as median [Q1, Q3], and were analyzed using the Kruskal–Wallis test. P-values < 0.05 were considered statistically significant.

Abbreviations: BMI, body mass index; CS, COVID-19-related stress; JST-IC, Japan Science and Technology Agency Index of Competence; LEH, lack of exercise habits; ODK, oral diadochokinesis; OHRQoL, oral health-related quality of life; Q1, first quartile; Q3, third quartile; TCI, tongue coating index.

### Poisson regression with robust standard errors

The prevalence ratios for poor OHRQoL based on the presence of CS and LEH are shown in Supplemental Tables S1 and S2 (available online). The presence of CS alone was not associated with poor OHRQoL (adjusted prevalence ratio [aPR]: 1.24, 95% CI: 0.85–1.81), whereas the presence of LEH alone showed a significant association with poor OHRQoL (aPR: 1.68, 95% CI: 1.12–2.51). Regarding the risk of poor OHRQoL, Group 4 (aPR: 2.20, 95% CI: 1.31–3.69) showed a significant association with poor OHRQoL, while Group 2 and Group 3 did not (Table 4). In addition, lower age (aPR: 0.97, 95% CI: 0.93 − 0.99), depressive tendencies (aPR: 2.45, 95% CI: 1.60–3.77), and fewer current teeth (aPR: 0.95, 95% CI: 0.93–0.97) were significantly associated with poor OHRQoL. Since the Lagrange multiplier test results for overdispersion with the null hypothesis that the auxiliary parameter of the negative binomial distribution is zero were not significant (Z = -6.19, p = 1.000), the Poisson regression with robust standard errors was considered reasonable.

**Table 4:** Adjusted prevalence ratios for poor OHRQoL.

Independent variable	n	Poor OHRQoL (%)	Crude PR	95% CI	Adjusted PR	95% CI
Age (years)			1.00	0.96 − 1.03	0.97	0.93 − 0.99
Sex (woman)	158	32.9	1.25	0.77 − 2.04	1.43	0.90 − 2.29
BMI (kg/m^2^)			1.02	0.96 − 1.08	1.01	0.95 − 1.06
JST-IC scores (score)			0.97	0.91 − 1.03	1.02	0.96 − 1.09
Depressive tendencies (presence)	23	65.2	2.41	1.65 − 3.51	2.45	1.60 − 3.77
Number of teeth (score)			0.96	0.94 − 0.98	0.95	0.93 − 0.97
Risk of poor OHRQoL						
Group 1: CS(-) , LEH(-)	86	25.6	1.00	(Reference)	1.00	(Reference)
Group 2: CS(-) , LEH( +)	32	34.4	1.34	0.74–2.45	1.25	0.68 − 2.30
Group 3: CS( +) , LEH(-)	70	27.1	1.06	0.63–1.80	1.00	0.61 − 1.65
Group 4: CS( +) , LEH( +)	27	55.6	2.17	1.33–3.56	2.20	1.31 − 3.69

Forced entry analysis.

Abbreviations: *BMI* body mass index; *CI* confidence interval; *CS* COVID-19-related stress; *JST-IC* Japan Science and Technology Agency Index of Competence; *LEH* lack of exercise habits; *OHRQoL* oral health-related quality of life; *PR* prevalence ratio.

## Discussion

The results of this cross-sectional study supported our hypothesis that the coexistence of stress, related to mobility restrictions and lifestyle changes during the COVID-19 pandemic, and LEH is associated with poor OHRQoL. Several studies have investigated the presence of subjective toothache during the COVID-19 pandemic through questionnaires^23^ and examined OHRQoL in patients who visited the dentist for acute symptoms^13^. However, no study has focused on the coexistence of CS and LEH in daily life. OHRQoL may decline because of organic factors such as decreased number of teeth and periodontal disease^24,25^ and functional factors such as occlusal force and masticatory function^24,26^, as well as psychological and social factors^8^. Furthermore, an association has been reported between OHRQoL and general health in older adults^27^. Our results suggest the need to consider not only the oral health status but also the psychosocial background, including stress and exercise habits of older adults, when designing oral healthcare and public health programs.

The COVID-19 pandemic has been reported to exacerbate feelings of depressed mood and helplessness in community-dwelling older people^6^. Anxiety and psychological distress due to COVID-19 were also found to be associated with poor OHRQoL^13^. However, in this study, CS alone was not associated with poor OHRQoL. One reason for the discrepancy between our results and those of previous studies may be that Iwamizawa City, where the survey was conducted, is a provincial city, and its environment was less susceptible to CS than large cities. Moreover, the number of COVID-19 cases in Iwamizawa City was zero in October when this survey was conducted^28^. Additionally, the participants voluntarily participated in health checkups; thus, many of them may have been active and health-conscious. Furthermore, the subjects' behavior may be influenced by their awareness of being in a study under observation^29^. Therefore, we created four groups defined by the presence or absence of CS and LEH and examined their association with poor OHRQoL in detail. Subsequently, Poisson regression with robust standard errors showed that the coexistence of CS and LEH was associated with poor OHRQoL. Furthermore, LEH alone was associated with poor OHRQoL; however, in Group 2, where CS was not present, LEH was not a risk factor for poor OHRQoL, suggesting that the coexistence of CS with LEH is an important factor for poor OHRQoL.

This is the first study to report an association between OHRQoL and LEH, although the underlying mechanism remains unclear. However, reports have shown that exercise can improve depressive and anxiety symptoms^30^, representing a potential alternative to antidepressant treatment in patients with depression^31^. The possible neurobiological mechanisms include an increase in brain-derived neurotrophic factors^32^ and the modulation of autonomic nervous system functions^33^. These findings suggest that exercise may affect OHRQoL by reducing stress. Exercise has been reported to prevent a decline in psychological well-being during the COVID-19 pandemic^34^. In this study, CS was possibly not alleviated due to LEH, and Group 4 showed an association with poor OHRQoL. In terms of the risk of poor OHRQoL, neither Group 2 nor Group 3 showed a significant association with poor OHRQoL in the Poisson regression with robust standard errors. In Group 2, CS was not present and thus did not need to be relieved by exercise; however, in Group 3, exercise habits were present, and CS was relieved by exercise. This may explain why no association with poor OHRQoL was found in these two groups. Additionally, the fact that CS alone was not associated with poor OHRQoL may have been influenced by the fact that of the 97 participants (Group 3 + Group 4) who experienced CS, 70 were in Group 3 and had exercise habits.

The Poisson regression with robust standard errors revealed an association between Group 4 and poor OHRQoL. In contrast, a simple comparison of the four groups found no difference in the proportion of patients with oral hypofunction. This suggests that the subjective assessment of OHRQoL by the General Oral Health Assessment Index (GOHAI) did not match the objective assessment of oral hypofunction. Previous studies have also reported discrepancies between subjective and objective assessments of masticatory function, which are associated with depressive symptoms, instrumental activities of daily living (IADL), and physical functions^35^. Another study reported that subjective xerostomia was associated with psychogenic factors and IADL, whereas objectively assessed hyposalivation was associated with being female and the use of gastrointestinal drugs^36^. From the above, it may be concluded that a decline in subjective oral health is related to IADL, physical functions, and psychological factors such as depressive symptoms. Additionally, in this study, the JST-IC scores were not associated with poor OHRQoL. This may be because many participants came to the wellness checkup site voluntarily, and their IADL and physical functions were relatively well-maintained. Therefore, in this study, poor OHRQoL may have been associated more with psychogenic factors, such as CS, than with IADL or physical functions.

Furthermore, the Poisson regression showed that lower age, depressive tendencies, and fewer current teeth were associated with poor OHRQoL, consistent with the results of previous studies^24,27^. However, the generalization and application of this study’s results should be conducted with care. All citizens aged ≥ 60 years were encouraged to participate in this survey, and individuals voluntarily participated in the wellness checkups. In other words, these participants may have had a high motivation for preserving their health, and many of them may have had high oral, physical, and cognitive functions. In terms of age group, more participants in this study were over 70 years old than the participants in the Survey of Dental Diseases^37^, a survey covering all of Japan (Supplemental Table S3).Nevertheless, the participants in our study had a higher mean JST-IC score than the standard value of 9.5^38^ and a low percentage of oral hypofunction (26.5%) compared with previous studies by Kugimiya et al. (43.6%)^39^ and Shimazaki et al. (62.9%)^40^. Therefore, sample bias must be considered when discussing the results, and random sampling with lower sample bias should be considered in the future. Additionally, given the rapid increase in the number of older adults over the age of 80^41^, considering these populations will be important for future studies. Furthermore, Iwamizawa City, the site of the survey, is a regional city. Previous studies have reported lower stress levels in rural areas than in large cities, where the cases of infection and deaths due to COVID-19 have been higher, owing to the high population density^42^. Iwamizawa City may, thus, have been an area with less CS. Finally, although a Japanese version of the Fear of COVID-19 Scale^43^ for assessing CS has now been developed and validated^44^, this version did not exist at the time this study began. Therefore, based on reports that mobility restrictions and lifestyle changes due to the COVID-19 pandemic are factors that induce stress^4^, we assessed CS by asking, “Do you feel stressed by the lifestyle changes and restrictions on going out due to the COVID-19 pandemic?” Furthermore, regarding the lack of exercise, a review^32^ that summarized the effects of exercise on anxiety symptoms in non-psychiatric patients highlighted the variations in the type and frequency of exercises in several studies. In our study, the type and intensity of exercise were not specified, but the frequency was set to at least once a week, based on a report suggesting that exercising once or twice a week is associated with lower mortality^45^.

This study has several limitations. First, since this was a cross-sectional study, the possibility of reverse causality cannot be ruled out. Second, although all citizens aged ≥ 60 years were invited to participate in the survey, the rate of participation was low because of the COVID-19 pandemic. Therefore, the sample size was not large enough to consider all the factors associated with OHRQoL, such as income and utilization of dental services^19,27^.

## Conclusion

In conclusion, we found that the coexistence of CS and LEH was associated with poor OHRQoL in community-dwelling older adults. Thus, CS and LEH may be associated with oral health in older adults independent of organic factors such as reduced number of teeth or functional factors such as occlusal force. Furthermore, OHRQoL is associated with general health in older adults. Therefore, CS and LEH are important factors to consider when designing both oral healthcare and public health programs.

## Methods

### Study design and participants

This was a cross-sectional study based on a survey on 34,564 community-dwelling older adults aged ≥ 60 years in Iwamizawa, a regional city in Hokkaido, northern Japan. This study included older adults who participated in wellness checkups in CHEER Iwamizawa (a research project titled “Checkup to discover HEalth with Energy for senior Residents” in Iwamizawa) in October 2020. The participants of CHEER Iwamizawa were recruited through a public relations magazine published by the city council and flyers posted at major public facilities in the city. In addition, local government employees visited older adults’ social clubs to describe the study’s purpose and recruit participants. The survey content was explained verbally and in writing to the participants, and written informed consent was obtained prior to the survey. This study was conducted in accordance with the Declaration of Helsinki and approved by the Ethics Committee of the Faculty of Dental Medicine, Hokkaido University (approval number: 2020–9).

### Evaluation of outcome

OHRQoL was evaluated using the Japanese version of the GOHAI^46^. The GOHAI consists of 12 questions rated on a five-point Likert scale assessing the frequency of oral health-related problems over the past three months. The total score ranges from 12 to 60; the higher the score, the higher the OHRQoL. The median scores, as per the GOHAI national norms for Japanese individuals, are 56.0 and 52.5 for men aged 60 − 69 years and 70 − 79 years, respectively, and 54.0 and 53.0 for women aged 60–69 years and 70 − 79 years, respectively. Therefore, in this study, participants scoring below these cutoff values were defined as the poor OHRQoL group, while the remaining participants were assigned to the good OHRQoL group.

### Defining the risk of poor OHRQoL

CS and LEH were defined as risk factors for poor OHRQoL, and their presence or absence was examined using self-administered questionnaires. CS was assessed by the question, “Do you feel stressed by the lifestyle changes and restrictions on going out due to the COVID-19 pandemic?” (Yes/To some extent/Not really/No). Participants who answered either *yes* or *to some extent* were considered to have CS. LEH was assessed by the question, “Do you perform exercises, such as walking, at least once a week?” (Yes/No). Participants who answered *no* were considered to lack exercise habits. Then, four mutually exclusive groups (risk of poor OHRQoL) were created based on the presence or absence of both CS and LEH (Group 1: no CS and no LEH; Group 2: no CS and LEH; Group 3: CS and no LEH; Group 4: both CS and LEH).

### Demographic characteristics

Data on participants’ age, sex, smoking status, body mass index, and medical history (malignant neoplasm, stroke, myocardial infarction, depression, and osteoarthritis) were collected using a self-administered questionnaire.

### Instrumental activities of daily living

IADL was assessed using the JST-IC^38^. The JST-IC consists of 16 questions with a “Yes/No” response format; the total score ranges from 0 to 16. No specific cutoff value was set; higher scores indicated higher IADL. The internal consistency obtained in the study population and example items of questionnaires are shown in Supplemental Table S4 (available online).

### Assessment of depressive tendencies

The Japanese version of the Geriatric Depression Scale short form (GDS-15) was used to assess depressive tendencies. The GDS-15 comprises 15 questions in a “Yes/No” response format, with the total score ranging from 0 to 15. In the present study, a total score of ≥ 6 indicated the presence of depressive tendencies^47^. The internal consistency obtained in the study population and example items of questionnaires are shown in Supplemental Table S4 (available online).

### Number of teeth

The number of teeth erupted in the oral cavity was recorded, excluding stump teeth and teeth with severe mobility.

### Diagnosis of oral hypofunction

Qualified examiners who received two hours of instruction and training from the authors (K.M. and Y.W.) regarding the appropriate data collection methods for measurements performed oral function measurements. However, interexaminer reliability measures were not obtained. There was no strict calibration of examiners for oral function measurement. However, this potential nondifferential misclassification would probably bias results toward the null hypothesis and not lead to overestimation of the observed associations. Significant robust associations were observed under these circumstances. The oral function was objectively assessed using six of the seven parameters previously described to diagnose oral hypofunction^48^: oral hygiene, oral moisture, occlusal force, tongue and lip movement, tongue pressure, and masticatory function (excluding swallowing function). Oral hygiene was assessed using the tongue coating index^49^ to determine the degree of tongue coating by visual inspection. Oral moisture was measured at the center of the tongue dorsum, approximately 10 mm from the apex of the tongue, using an oral moisture checker (Mucus, Life Co., Ltd., Saitama, Japan). Each measurement was taken thrice, and the median value was used. The occlusal force of the entire dentition was measured using a pressure-indicating film (Dental Prescale II, GC Corp, Tokyo, Japan) during three seconds of clenching in the intercuspal position. For denture users, the occlusal force was measured with their dentures in place. Tongue and lip movements were assessed using oral diadochokinesis. Participants were asked to repeat the syllables /pa/, /ta/, and /ka/ for five seconds, and the number of each syllable pronounced per second was counted using an automatic counter (Kenkoukun Handy, Takei Scientific Instruments Co., Ltd., Niigata, Japan). Tongue pressure was measured thrice using a tongue pressure measuring instrument (JMS tongue pressure measuring instrument, JMS Co., Ltd., Hiroshima, Japan), and the maximum value was used. The masticatory function was measured using a masticatory ability testing system (Glucosensor GS-II, GC Corp., Tokyo, Japan).

Using previously established guidelines^48^, the cutoff values for the above six items were as follows: tongue coating index ≥ 50%; oral moisture < 27.0; occlusal force < 500 N; any of the /pa/, /ta/, or /ka/ syllables repeated < 6 times/second for diadochokinesis; tongue pressure < 30 kPa; and masticatory function < 100 mg/dL. Oral hypofunction was defined when at least three of the six measurements met these criteria.

### Sample size calculation

Sample size calculation was performed using G*Power 3.1.9.7^50^. Assuming a two-tailed Mann–Whitney U test with α = 0.05 and d = 0.50, an estimated 134 participants were required to achieve a power of 0.80.

### Statistical analyses

Descriptive statistics, comparisons between the two groups with and without CS, and comparisons between the two groups with and without LEH were conducted by performing Mann–Whitney U tests for continuous variables and the chi-square test or Fisher’s exact test for categorical variables. The scoring of categorical valuables is shown in Supplemental Table S5 (available online). Comparisons among the four groups of risk factors for poor OHRQoL were made by performing the Kruskal–Wallis test for continuous variables and the chi-square test or Fisher’s exact test for categorical variables. Thereafter, Poisson regression with robust standard errors^51^ was used to calculate the prevalence ratio for poor OHRQoL of CS alone and LEH alone, respectively. Adjustment factors included sociodemographic characteristics (i.e., age, sex, body mass index, JST-IC), depressive tendencies^27^, and the number of teeth^24^. Subsequently, the prevalence ratio for poor OHRQoL regarding the risk factors of poor OHRQoL was calculated. When the study design is similar to the present study, the odds ratio is often obtained by logistic regression analysis. However, in logistic regression analysis, the odds ratio is known to deviate from the true relative risk as the frequency of the outcome increases, overestimating the odds ratio when the risk ratio is greater than 1.0 and underestimating the odds ratio when the risk ratio is less than 1.0. ^52^. Therefore, in this study, Poisson regression with robust standard errors was used to calculate estimates that approximate the true relative risk. All analyses were performed using SPSS Statistics version 27 (IBM Corp., Armonk, NY, USA), and the significance level (two-tailed) was set at 5%. We followed the Strengthening the Reporting of Observational Studies in Epidemiology (STROBE) guidelines.

## Supplementary Information

Supplementary Information.

## Data Availability

The data used in this study are available from the corresponding author upon reasonable request.
