# Supplementary material for: COVID-19-related stress, exercise, and oral health-related quality of life among community-dwelling older adults who participated in the CHEER Iwamizawa project, Japan
Source: Sci Rep. 2022 Nov 27;12:20347. doi: 10.1038/s41598-022-24806-1 (PMC9701693; doi:10.1038/s41598-022-24806-1)
Supplement: Supplementary file 1 — Supplementary Information. [file 41598_2022_24806_MOESM1_ESM.pdf]

Supplemental Figure and Table

COVID-19-related stress, exercise, and oral health-related quality of life among community-dwelling older adults who participated in the CHEER Iwamizawa project, Japan

Kazuhito Miura, Yutaka Watanabe, Haruhisa Baba, Kimiya Ozaki, Takae Matsushita, Miyako Kondoh, Kazutaka Okada, Shinji Nakaoka, Katsuhiko Ogasawara, Teppei Suzuki, Hiroshi Saito, Takashi Kimura, Akiko Tamakoshi, and Yutaka Yamazaki

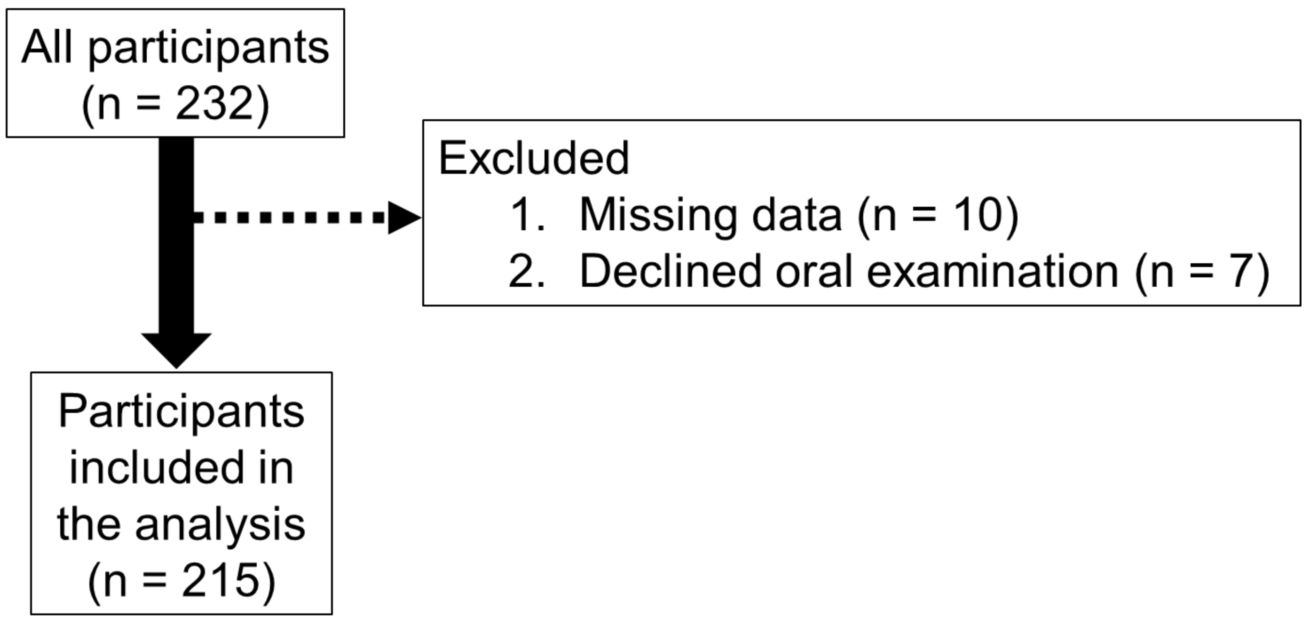

Supplemental Figure S1. Flowchart of the participant selection process

The arrow with dotted lines indicates the participants excluded from the analysis. The downward arrow indicates the participants included in the analysis.

Supplemental Table S1. Adjusted prevalence ratios of poor OHRQoL for participants with COVID-19-related stress

Independent variable	n	Poor OHRQoL (%)	Crude PR	95% CI	Adjusted PR	95% CI
CS (presence)	97	35.1	1.25	0.84–1.86	1.24	0.85–1.81

Note: Forced entry analysis. Adjusted prevalence ratios were adjusted for age, sex, body mass index, Japan Science and Technology Agency Index of Competence score, depressive tendencies, and the number of teeth.

Abbreviations: CI, confidence interval; CS, COVID-19-related stress; OHRQoL, oral health-related quality of life; PR, prevalence ratio.

Supplemental Table S2. Adjusted prevalence ratios of poor OHRQoL for participants with a lack of exercise habits

Independent variable	n	Poor OHRQoL (%)	Crude PR	95% CI	Adjusted PR	95% CI
LEH (presence)	59	44.1	1.68	1.14–2.48	1.68	1.12–2.51

Note: Forced entry analysis. Adjusted prevalence ratios have been adjusted for age, sex, body mass index, Japan Science and Technology Agency Index of Competence score, depressive tendencies, and the number of teeth. Abbreviations: CI, confidence interval; LEH, lack of exercise habits; OHRQoL, oral health-related quality of life; PR, prevalence ratio.

Supplemental Table S3. Comparison of age and sex in the Survey of Dental Diseases and this study

	Survey of Dental Diseases (2016) n=1913		CHEER Iwamizawa (2020) n=215	
	n	%	n	%
Age				
60～64	351	18.3	13	6.0
65～69	503	26.3	33	15.3
70～74	380	19.9	69	32.1
75～79	319	16.7	57	26.5
80～84	224	11.7	33	15.3
85～	136	7.1	10	4.7

Supplemental Table S4. The internal consistency obtained in the study population and example items of questionnaires

Questionnaire	Items
GDS-15 (Cronbach's α = 0.78)	1. Are you basically satisfied with your life?<br>2. Have you dropped many of your activities and interests?<br>3. Do you feel your life is empty?<br>4. Do you often get bored?<br>5. Are you in good spirits most of the time?<br>6. Are you afraid that something bad is going to happen to you?<br>7. Do you feel happy most of the time?<br>8. Do you feel helpless?<br>9. Do you prefer to stay at home, rather than going out and doing new things?<br>10. Do you feel you have more problems with your memory than most?<br>11. Do you think it is wonderful to be alive?<br>12. Do you feel pretty worthless the way you are now?<br>13. Do you feel full of energy?<br>14. Do you feel your situation is hopeless?<br>15. Do you think that most people are better off than you are?
JST-IC (Cronbach's α = 0.77)	1. Can you use a mobile phone?<br>2. Can you use the ATM?<br>3. Can you operate a video recorder such as a Blu-ray recorder or DVD player?<br>4. Can you send an e-mail using a mobile phone or computer?<br>5. Are you interested in news and events from overseas?<br>6. Can you determine the credibility of health-related information?<br>7. Do you enjoy art, films, or music?<br>8. Do you watch educational/cultural programs?<br>9. Do you follow any measures to prevent yourself from becoming a victim of crimes?<br>10. Do you try to be creative while doing daily tasks (i.e., cleaning, cooking)?<br>11. Can you take care of an ill person?<br>12. Do you take care of your grandchildren, family members, or acquaintances?<br>13. Do you participate in regional festivals or events?<br>14. Do you participate in a neighborhood association or a residents' association?<br>15. Would you be able to assume a managerial position such as an organizer in a residents' association or group activities?<br>16. Do you engage in charity or volunteer activities?

Supplemental Table S5. Scoring of categorical valuables

Variable	Scoring
Women, n (%)	Yes=1, No=0
Current smokers, n (%)	Yes=1, No=0
Medical history	
Malignant neoplasm	Yes=1, No=0
Stroke	Yes=1, No=0
Myocardial infarction	Yes=1, No=0
Depression	Yes=1, No=0
Osteoarthritis	Yes=1, No=0
Depressive tendencies	GDS-15 score ≥6=1, <6=0
Oral hypofunction	the six measurements met the criteria (tongue coating index ≥50 %; oral moisture <27.0; occlusal force <500 N; any of the /pa/, /ta/, or /ka/ syllables repeated <6 times/second for diadochokinesis; tongue pressure <30 kPa; and masticatory function <100 mg/dL) ≥3=1, <3=0
Risk of poor OHRQoL	Group 1: no CS and no LEH=0<br>Group 2: no CS and LEH=1<br>Group 3: CS and no LEH=2<br>Group 4: both CS and LEH=3
OHRQoL	Below the median scores of the GOHAI national norms for Japanese individuals=1<br>Above the median scores of the GOHAI national norms for Japanese individuals=0<br>(The median scores, as per the GOHAI national norms for Japanese individuals, are 56.0 and 52.5 for men aged 60−69 years and 70−79 years, respectively, and 54.0 and 53.0 for women aged 60–69 years and 70−79 years, respectively.)

Abbreviations: CS, COVID-19-related stress; GOHAI, General Oral Health Assessment Index; LEH, Lack of exercise habits; OHRQoL, Oral health-related quality of life.
